# Assessment of the Activity of Tigecycline against Gram-Positive and Gram-Negative Organisms Collected from Italy between 2012 and 2014, as Part of the Tigecycline Evaluation and Surveillance Trial (T.E.S.T.)

**DOI:** 10.3390/ph9040074

**Published:** 2016-11-26

**Authors:** Stefania Stefani, Michael J. Dowzicky

**Affiliations:** 1 Department of Bio-Medical Sciences, University of Catania, Via Androne 81, Catania 95124, Italy; 2 Pfizer Inc., Collegeville, PA 19426, USA; michael.dowzicky@pfizer.com

**Keywords:** Gram-positive, Gram-negative, antimicrobial resistance, Italy, multidrug resistance, surveillance, tigecycline

## Abstract

As part of the Tigecycline Evaluation and Surveillance Trial (T.E.S.T) we report the in vitro activity of tigecycline and its comparators against Gram-negative and Gram-positive organisms collected from Italian centers between 2012 and 2014. Minimum inhibitory concentrations were determined according to the broth microdilution methodology of the Clinical and Laboratory Standards Institute, and antimicrobial resistance was determined using the European Committee on Antimicrobial Susceptibility Testing interpretive criteria. Among the Enterobacteriaceae, 31% of *Escherichia coli* isolates, 22% of *Klebsiella pneumoniae*, and 1% of *Klebsiella oxytoca* were extended-spectrum β-lactamase producers (ESBLs). Resistance rates among ESBL-*K*. *pneumoniae* and ESBL-*E. coli* to meropenem were 24% and <1%, respectively. Thirty-seven percent of *K. pneumoniae* were multidrug resistant (MDR) strains. Resistance rates among isolates of *Acinetobacter baumannii* to amikacin, levofloxacin and meropenem were between 84% and 94%. Eighty percent of *A. baumannii* isolates were MDR strains. Methicillin-resistant *Staphylococcus aureus* (MRSA) accounted for 38% of *S. aureus* isolates. No isolates of MRSA were resistant to linezolid, tigecycline or vancomycin. Antimicrobial resistance remains a problem in Italy with increasing numbers of MDR organisms. Despite high levels, MRSA rates appear to be stabilising. Tigecycline retains its in vitro activity against the majority of organisms, including those with multidrug resistance.

## 1. Introduction

Across Europe the overuse and misuse of antibiotics has led to increasing rates of antimicrobial resistance, particularly in the southern and eastern areas [1]. Contributing factors include varying rates of infection control, incorrect prescribing behavior, and a lack of patient knowledge and understanding [2,3]. In recent years many healthcare facilities in Europe have implemented infection control programs to combat antimicrobial resistance [1]. Following this, rates of methicillin-resistant *Staphylococcus aureus* (MRSA) have stabilized in some areas and have decreased in others [1]. Italy is no exception to this, with reports of decreasing rates of MRSA over recent years [4].

A recent report by the European Antimicrobial Resistance Surveillance Network (EARS-Net) showed that in Italy there were increasing rates of resistance among isolates of *Klebsiella pneumoniae*, *Escherichia coli* and *Acinetobacter* spp. to a range of antimicrobial agents, including fluoroquinolones, third-generation cephalosporins, aminoglycosides and carbapenems, alone, or in combination [1]. This rise in multidrug resistance has increased the use of carbapenems resulting in escalating numbers of carbapenem-resistant bacteria [5]. Rates of penicillin- and macrolide-non-susceptibility among *Streptococcus pneumoniae* also remain high in Italy [1].

Tigecycline is a broad-spectrum antimicrobial agent with in vitro activity against Gram-positive, Gram-negative and multidrug-resistant (MDR) pathogens. It is licensed to treat complicated skin and skin structure infections and complicated intra-abdominal infections in the USA and Europe, and also community-acquired bacterial pneumonia in the USA [6]. The Tigecycline Evaluation and Surveillance Trial (T.E.S.T.) is a global surveillance study which has been ongoing since 2004 and monitors the in vitro antibacterial activity of tigecycline and comparator agents against a range of clinically important Gram-positive and Gram-negative organisms. This paper serves as an update of the one by Stefani et al., which covered the period 2004 to 2011 [7]. Here, we report on antimicrobial resistance among isolates of Gram-positive and Gram-negative organisms and their resistant phenotypes collected from Italian T.E.S.T. centers between 2012 and 2014. We also discuss the 2012 to 2014 data in comparison to the 2004 to 2011 data published in the earlier paper by Stefani et al. [7].

## 2. Results

Between 2012 and 2014 a total of 6605 isolates were examined as part of the T.E.S.T. Italy study; 4535 Gram-negative and 2070 Gram-positive isolates.

### 2.1. Gram-Negative Organisms

Among isolates of *K. pneumoniae*, 52%–57% were resistant to amoxicillin/clavulanate, cefepime, ceftriaxone, or levofloxacin (Table 1). Resistance to amikacin, meropenem and piperacillin/tazobactam decreased by 10%–13% between 2012 and 2014. Resistance to tigecycline was seen among 6% of *K. pneumoniae* isolates. Among *Klebsiella oxytoca* isolates, resistance to amoxicillin/clavulanate, ceftriaxone or piperacillin/tazobactam decreased by 15%–21% between 2012 and 2013, and 17%–26% between 2012 and 2014. Although, *K. oxytoca* isolates resistant to levofloxacin, cefepime and meropenem were detected in 2012 and 2013 no resistant isolates were detected in 2014. Between 2012 and 2014 only one *K. oxytoca* isolate was resistant to tigecycline.

Resistance among isolates of *E. coli* to the majority of antimicrobials in the T.E.S.T. panel remained consistent between 2012 and 2014 (Table 1). Resistance was highest to ampicillin (77%). No *E. coli* isolates were resistant to tigecycline and only one isolate (collected in 2013) was resistant to meropenem.

Isolates of *Enterobacter* spp. and *Serratia marcescens* show similar results; ≤5% of isolates were resistant to amikacin, meropenem or tigecycline (Table 1). Among isolates of *Enterobacter* spp., percentage resistance to cefepime, ceftriaxone and levofloxacin was ≥10% higher than that for *S. marcescens*. Twenty-nine percent of *Enterobacter* spp. and 8% of *S. marcescens* were resistant to piperacillin/tazobactam.

Breakpoints were only available for three of the antimicrobials in the T.E.S.T. panel against *A. baumannii*: amikacin, levofloxacin, and meropenem. Between 2012 and 2014 resistance to each of these antimicrobials was ≥84% (Table 1). Resistance among *Psuedomonas aeruginosa* isolates to amikacin, cefepime, ceftazidime, levofloxacin, meropenem or piperacillin/tazobactam remained below 41% in 2012, 2013 and 2014.

Between 2012 and 2014, ≤10% of *Haemophilus influenzae* isolates were resistant to amoxicillin/clavulanate, ampicillin and ceftriaxone (Table 1). Less than 1% of isolates were resistant to levofloxacin, meropenem and minocycline.

### 2.2. Gram-Negative Phenotypes

Between 2012 and 2014, 22% of *K. pneumoniae* were extended-spectrum β-lactamase (ESBL)-producers and 37% were MDR (Table 2). High percentages of resistance among ESBL-*K. pneumoniae* were reported for ceftriaxone (99%) and cefepime (93%) (Table 3). Resistance to amikacin, amoxicillin/clavulanate and meropenem decreased by 10%–12% between 2012 and 2014. Resistance to piperacillin/tazobactam reduced from 70% in 2012 to 44% in 2014. Between 2012 and 2014 a 10% increase in resistance among ESBL-*K. pneumoniae* to cefepime was seen. Tigecycline showed the lowest percentage resistance (8%) between 2012 and 2014. Between 2012 and 2014 ≥94% of MDR *K. pneumoniae* isolates were resistant to amoxicillin/clavulanate, cefepime, ceftriaxone, levofloxacin and piperacillin/tazobactam; 88% of isolates were resistant to meropenem. Twelve percent of MDR *K. pneumoniae* were resistant to tigecycline.

Thirty-one percent of *E. coli* isolates collected between 2012 and 2014 were ESBL-producers (Table 2). Resistance among ESBL-producing *E. coli* to the majority of antimicrobials in the T.E.S.T. panel remained constant between 2012 and 2014 (Table 3). One exception to this was amoxicillin/clavulanate, which showed a decrease in resistance from 71% in 2012, to 55% in 2013, and 65% in 2014. Cefepime resistance also decreased from 88% in 2012 to 67% in 2014. No isolates of ESBL-*E. coli* were resistant to tigecycline.

Of the *A. baumannii* isolates submitted between 2012 and 2014, 80% were MDR strains (Table 2). Among *P. aeruginosa*, 19% of isolates were MDR strains (Table 2). Among MDR *P. aeruginosa*, ≥82% of isolates were resistant to cefepime, levofloxacin, meropenem and piperacillin/tazobactam. Resistance among MDR *P. aeruginosa* to amikacin fluctuated from 51% in 2012, to 44% in 2013 and increased to 61% in 2014. Resistance to ceftazidime decreased by 15% between 2012 and 2014 (Table 3).

Fewer than 10 isolates of ESBL-*K. oxytoca*, β-lactamase positive *H. influenzae* (βLPos *H. influenzae*) and β-lactamase negative ampicillin-resistant *H. influenzae* (BLNAR *H. influenzae*) were submitted in any one year between 2012 and 2014 (Table 2).

### 2.3. Gram-Positive Organisms

Among *S. aureus* isolates, 62% were methicillin-susceptible (MSSA) and 8% of these isolates were resistant to levofloxacin and 2% were resistant to minocycline (Table 4). No MSSA isolates were resistant to linezolid, tigecycline and vancomycin.

No *Enterococcus faecalis* isolates were resistant to ampicillin, linezolid or tigecycline (Table 4). Among *E. faecium* isolates, 86% were resistant to ampicillin. No isolates of *E. faecium* were resistant to linezolid and only one isolate (in 2013) was resistant to tigecycline.

Among *S. pneumoniae* isolates the highest rates of resistance were to the macrolides (36%–37%) (Table 4). Between 2012 and 2014 resistance to azithromycin, erythromycin and clarithromycin decreased by 16%–17%. Resistance to clindamycin reduced from 36% in 2012 to 26% in 2014. No isolates of *S. pneumoniae* were resistant to ceftriaxone, linezolid, meropenem or vancomycin.

Between 2012 and 2014, 84% of *Streptococcus agalactiae* isolates were resistant to minocycline, 2% were resistant to levofloxacin; no isolates were resistant to linezolid, penicillin, tigecycline or vancomycin (Table 4).

### 2.4. Gram-Positive Phenotypes

**Figure 1:**
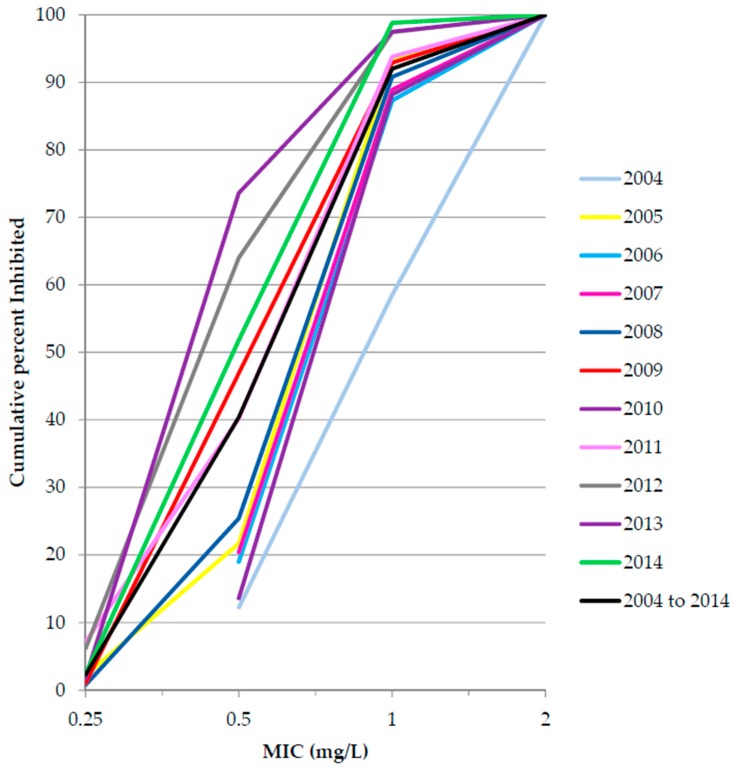
Cumulative minimum inhibitory concentration (MIC) distribution of vancomycin against methicillin-resistant *Staphylococcus aureus*, Italy 2004 to 2014.

Methicillin-resistant *S. aureus* accounted for 38% of *S. aureus* isolates collected between 2012 and 2014 (Table 2). No isolates of MRSA were resistant to linezolid, tigecycline or vancomycin; 83% of isolates were resistant to levofloxacin (Table 5). Data analysis of vancomycin against MRSA showed a downward shift in minimum inhibitory concentration (MICs) between 2004 and 2014 (Figure 1). Between 2012 and 2014, 20% of *E. faecium* isolates were vancomycin-resistant (Table 2); none of these isolates were resistant to linezolid or tigecycline (Table 5). Ten isolates of vancomycin-resistant *E. faecalis* and six isolates of penicillin-resistant *S. pneumoniae* were identified between 2012 and 2014 (Table 2).

## 3. Discussion

Italy has relatively high rates of antimicrobial resistance compared to other parts of Europe [1]. Reports show that despite an improvement in infection control in Italy, more effort is needed to standardize infection control procedures between regions and hospitals, as well as to ensure their effective operation [9,10]. This report on T.E.S.T. data from Italy between 2012 and 2014 is an update of the previous publication by Stefani et al. which presented data from the 2004 to 2011 time period [7]. Comparisons between the two studies are limited because the current report uses the European Committee on Antimicrobial Susceptibility Testing (EUCAST) criteria for determining susceptibility and resistance and the previous publication used the Clinical and Laboratory Standards Institute (CLSI) interpretive criteria [8,11]. Both guidelines use different methods for determining clinical breakpoints: CLSI use a variant of the error-rate-bounded method which incorporates an intermediate zone [12], whereas EUCAST define MIC breakpoints on the basis of epidemiological cut-off values, pharmacokinetic/pharmacodynamic parameters, and by correlating MIC breakpoints to zone diameter values using the “MIC-coloured zone diameter histogram technique” [13]. EUCAST breakpoints do not define an intermediate category which Marchese et al. [14], and Hombach et al. [15,16] conclude will lead to increasing numbers of resistant bacteria being reported in countries that shift from using CLSI to EUCAST criteria, such as Italy. Generally, for the organisms included in this study breakpoints are different between CLSI and EUCAST, with EUCAST typically having lower susceptibility breakpoints. The decision was taken to use the EUCAST criteria in this report as these breakpoints are now considered the European standard and their use would allow the comparison of data from this study with other contemporary studies, it was also felt that the data would be more meaningful to healthcare providers currently practicing.

Our report shows comparable rates of ESBL-producing *K. pneumoniae* between 2012 and 2014 to that reported in the earlier study by Stefani et al. (22% and 24% respectively) [7]. However, the number of ESBL-producing *E. coli* isolates has increased between the two studies, from 25% (2004–2011), to 31% (2012–2014) [7]. Tigecycline and meropenem were the most active agents against *E. coli* and its resistant phenotype (≥98% susceptible); this result is comparable with that by Stefani et al. [7]. A surveillance study by Jones et al. monitored antimicrobial resistance in 21 European countries, including Italy, in 2011. Similarly they also reported elevated levels of ESBL-producing *E. coli* and *Klebsiella* spp. (20.1% and 45.7%, respectively) in Europe, as well as comparable rates of susceptibility among ESBL-*E. coli* to tigecycline and carbapenems (>99%) [17].

Increasing numbers of carbapenem-resistant Enterobacteriaceae are a major global health concern and Italy has one of the highest levels of carbapenem resistance in Europe [1,5,17,18,19,20]. A survey conducted in Italy by Giani et al. in 2011 identified that 2% of all Enterobacteriaceae were carbapenem-resistant, and the majority of these were *K. pneumoniae* (86.7%) [5]. In 2012 the European Center for Disease Prevention and Control (ECDC) launched the European survey of carbapenemase-producing Enterobacteriaceae (EuSCAPE) which aimed to monitor epidemiology, undertake surveillance and enhance laboratory capacity and diagnostics [20,21]. A recent report by Albiger et al. on data collected from the EuSCAPE project in 2015 identified that Italy was one of four European countries that classified carbapenemase-producing Enterobacteriaceae as an endemic situation [20]. A Spanish study by Palacios-Baena et al. also reported that *K. pneumoniae* isolates accounted for a large proportion of carbapenemase-producing Enterobacteriaceae (74%) [22]. Our report shows similar results; in total we report 8.8% (268/3053) of Enterobacteriaceae isolates were meropenem-resistant; 93.3% (250/268) of these were *K. pneumoniae* isolates.

Jones et al. reported increasing rates of carbapenem resistance among *Klebsiella* spp. in 2011 from Bulgaria, Greece, Israel, Italy, Poland, Romania, Russia and Turkey [17]. The results from the recent EARS-Net report show that in 2014, Greece, Italy and Romania had the highest levels of carbapenem resistance among *K. pneumoniae* isolates (62.3%, 32.9% and 31.5%, respectively). Other European countries report <10% of *K. pneumoniae* isolates were resistant to carbapenems [1]. A report by Magiorakos et al. on invasive *K. pneumoniae* isolates collected as part of the EARS-Net study between 2005 and 2010 identified there were 18 European countries that reported at least one carbapenem-resistant *K. pneumoniae* isolate [19]. In 2010 Greece reported the highest rate of carbapenem-resistant *K. pneumoniae* (49.8%), followed by Cyprus (16.4%), Italy (12.5%), Hungary (5.9%) and Portugal (2.2%) [19]. Percentages of carbapenem-resistant *K. pneumoniae* continue to increase in Italy, from 15% in 2010, to 27% in 2011 and up to 33% in 2014 [1,23]. This is consistent with our report which shows 33% of *K. pneumoniae* isolates were resistant to meropenem between 2012 and 2014.

The previous paper by Stefani et al. did not present data on MDR *K. pneumoniae* [1]. In our analysis, rates of meropenem resistance among MDR *K. pneumoniae* were 86%–90% between 2012 and 2014. However, statistical analysis using the Cochrane-Armitage trend test shows a significant (*p* < 0.0001) increase in resistance to meropenem among MDR *K. pneumoniae* isolates collected between 2004 and 2014; 4.5% (2/44) in 2008, 46.7% (14/30) in 2009, 60% (42/70) in 2010 to 89.4% (101/113) in 2011. These results must be treated with caution due to low *n* values, although this demonstrates how quickly antimicrobial resistance can become a problem. Conversely, rates of resistance to amikacin among MDR *K. pneumoniae* decreased significantly (*p* < 0.0001) over the course of the study from 68.8% (11/16) in 2006 to 21.1% (12/57) in 2014.

*Acinetobacter baumannii* and *P.aeruginosa* and their MDR strains are serious nosocomial pathogens and are intrinsically resistant to many antimicrobials [24,25]. Isolates of *Acinetobacter* spp. are reported more frequently in eastern and southern areas of Europe [1]. Our report identifies 80% of *A. baumannii* isolates collected in Italy were MDR; this is comparable with results from EARS-Net (>87% of *Acinetobacter* spp. from Italy were resistant to fluoroquinolones, aminoglycosides and/or carbapenems) [1]. Tigecycline remains active against *A. baumannii* with an MIC_90_ of 2 mg/L, which is comparable with data reported by Stefani et al. [7], as well as those from other Italian studies by Mezzatesta et al. and Jones et al. [17,26].

Our report shows that rates of MRSA remain high in Italy (2012–2014, 38% of *S. aureus* isolates were methicillin-resistant) however rates appear to be stabilizing. Rates of MRSA fluctuated between 34% and 41% between 2012 and 2014 and these results are comparable with those previously published by Stefani et al. [7]. Similarly, an Italian study by Campanile et al. in 2012 identified 35.8% of *S. aureus* isolates as MRSA [4]. The EARS-Net report shows that Italy was one of seven European countries to have percentages of MRSA >25%. However, there was a decrease in rates of MRSA in Italy between 2011 and 2014 (38.2% and 33.6%, respectively) [1].

As with other studies, both from Italy and other European countries, linezolid, tigecycline and vancomycin were the most active agents against *S. aureus*, including MRSA [7,17,27,28]. There were no MRSA or enterococci isolates that were resistant to linezolid despite other European reports showing outbreaks of linezolid-resistant MRSA and linezolid-resistant enterococci [17,29,30,31]. Vancomycin-resistant *S. aureus* remains rare with only a few cases reported globally, mainly in the USA [32]; however there has been a recent report of the first vancomycin-resistant *S. aureus* strain in Portugal [33]. A cumulative plot of *S. aureus* MICs over time showed a downward shift in MICs in Italy between 2004 and 2014. Several studies have evaluated vancomycin MIC creep (defined as the progressive increase in vancomycin MICs within a susceptible range) in *S. aureus* over time [34,35,36]; however our study appears to shown the opposite, with MICs decreasing.

Rates of vancomycin-resistant *E. faecalis* remain low (3%), with less than 10 isolates submitted in any one year, which is comparable with other parts Europe [1]. Our report shows that 20% of *E. faecium* isolates were vancomycin-resistant. However, EARS-Net reported lower percentages of vancomycin-resistant *E. faecium* in Italy (8.5% in 2014) [1].

Tigecycline retained good in vitro activity, with low rates of resistance among Gram-positive (<1%) and Gram-negative organisms (≤6%). Resistance rates among Gram-positive organisms to tigecycline are similar to that reported by Stefani et al. and Jones et al. [7,17]. Similar to our study, Jones et al. use EUCAST breakpoints to determine antimicrobial resistance [17]. Both reports identify there were no isolates of *E. coli* resistant to tigecycline. Our study reports marginally higher rates of resistance among *K. pneumoniae* (6%) and *Enterobacter* spp. (5%) isolates to tigecycline compared to that reported by Jones et al. for *Klebsiella* spp. (1%) and *Enterobacter* spp. (1.2%) [17]. Tigecycline is not active in vitro against *P. aeruginosa*. We report that 90% of *A. baumannii* isolates were inhibited by tigecycline at a concentration of 2 mg/L. This is comparable with reports from Italy [26], Eastern Europe [27] and Spain [28].

Limitations of this study include the varying number of participating centers and isolates submitted between years, which may cause fluctuations in antimicrobial resistance. It should be noted that despite efforts of Magiorakos et al. to standardize methodology to define MDR, variations in definitions between studies exists, which may cause limitations when comparing rates of MDR between studies [37]. We report the in vitro activity of antimicrobial agents, which limits the ability to compare the relationship of serum levels to dose and resistance at the site of infection.

This report shows that antimicrobial resistance in Italy continues to be major public health concern. There are increasing numbers of MDR organisms, particularly MDR *A. baumannii*. Despite levels of MRSA remaining high, this appears to have stabilized over recent years. The escalation of carbapenem-resistant Enterobacteriaceae in Italy gives cause for concern and it is therefore essential to monitor these organisms. Tigecycline continues to retain its in vitro activity against the majority of organisms including those with multidrug resistance. The results of this study show the importance of continuing surveillance of antimicrobial resistance and susceptibility to help to reduce the incidence of infection and optimize the use of antimicrobial agents.

## 4. Materials and Methods

Between 2012 and 2014 a total of 19 Italian centers submitted isolates as part of the T.E.S.T. study. All centers did not participate in all years. Ten centers participated for 3 years, four centers for 2 years, and five centers for 1 year. Details of the isolate collection and antimicrobial susceptibility testing have been previously published (e.g., Stefani et al. [7]). Minimum inhibitory concentrations were determined using broth microdilution methodology described by the CLSI [38]. Antimicrobial susceptibility was determined according to EUCAST interpretive criteria [8]. Methicillin resistance in *S. aureus* and ESBL-production among *E. coli* and *Klebsiella* spp. were determined by IHMA according to CLSI guidelines [39].

In this study multidrug resistance was defined as resistance to three or more classes of antimicrobial agents. Classes used to define MDR *Acinetobacter baumannii* and *P. aeruginosa* were the same as previously described by Stefani et al. [7]. Classes used to define MDR *K. pneumoniae* were aminoglycosides (amikacin), β-lactams (ampicillin, amoxicillin/clavulanate, cefepime, ceftriaxone or piperacillin/tazobactam), carbapenems (imipenem/meropenem), glycylcycline (tigecycline) and quinolones (levofloxacin).

## Figures and Tables

**Table 1:** Antimicrobial activity among Gram-negative organisms collected in Italy between 2012 and 2014.

	2012				2013				2014				2012–2014			
	MIC_50_	MIC_90_	%S	%R	MIC_50_	MIC_90_	%S	%R	MIC_50_	MIC_90_	%S	%R	MIC_50_	MIC_90_	%S	%R
***Klebsiella pneumoniae***																
	*N* = 297				*N* = 304				*N* = 154				*N* = 755			
AMK	4	32	56.2	21.2	2	32	73.7	11.2	4	16	70.8	9.1	4	32	66.2	14.7
AMC	32	≥64	38.0	62.0	16	≥64	46.1	53.9	16	≥64	44.2	55.8	16	≥64	42.5	57.5
FEP	32	≥64	42.4	55.2	2	≥64	48.7	48.0	16	≥64	42.2	55.8	16	≥64	44.9	52.5
CRO	64	64	40.4	58.9	8	64	48.7	51.3	32	64	42.2	57.1	32	64	44.1	55.5
LVX	≥16	≥16	39.7	58.6	2	≥16	48.7	49.0	8	≥16	42.9	56.5	8	≥16	44.0	54.3
MEM	0.12	≥32	57.2	41.4	≤0.06	≥32	70.7	25.7	≤0.06	≥32	68.2	31.8	0.12	≥32	64.9	33.1
MIN	4	8	-	-	2	16	-	-	2	8	-	-	4	8	-	-
TZP	128	≥256	43.1	54.5	8	≥256	53.3	40.8	8	≥256	53.9	41.6	16	≥256	49.4	46.4
TGC	1	2	82.2	4.7	0.5	2	79.6	6.3	1	2	76.6	6.5	1	2	80.0	5.7
***Klebsiella oxytoca***																
	*N* = 54				*N* = 73				*N* = 26				*N* = 153			
AMK	2	4	98.1	0.0	2	4	98.6	0.0	2	2	100	0.0	2	4	98.7	0.0
AMC	4	≥64	70.4	29.6	2	8	91.8	8.2	2	4	96.2	3.8	2	32	85.0	15.0
FEP	≤0.5	4	81.5	9.3	≤0.5	≤0.5	95.9	2.7	≤0.5	≤0.5	100	0.0	≤0.5	1	91.5	4.6
CRO	≤0.06	16	79.6	20.4	≤0.06	0.25	93.2	5.5	≤0.06	0.12	96.2	3.8	≤0.06	4	88.9	10.5
LVX	0.06	8	88.9	11.1	0.03	0.12	94.5	2.7	0.03	0.06	100	0.0	0.03	0.25	93.5	5.2
MEM	≤0.06	0.12	98.1	1.9	≤0.06	≤0.06	98.6	1.4	≤0.06	≤0.06	100	0.0	≤0.06	0.12	98.7	1.3
MIN	1	2	-	-	1	4	-	-	1	1	-	-	1	2	-	-
TZP	2	≥256	75.9	24.1	1	4	97.3	2.7	1	2	96.2	3.8	1	32	89.5	10.5
TGC	0.25	0.5	98.1	0.0	0.25	1	94.5	1.4	0.25	0.5	100	0.0	0.25	0.5	96.7	0.7
***Escherichia coli***																
	*N* = 332				*N* = 428				*N* = 226				*N* = 986			
AMK	2	8	97.0	0.9	2	8	95.8	1.4	2	8	95.6	1.3	2	8	96.1	1.2
AMC	8	32	53.6	46.4	8	32	61.0	39.0	8	32	59.3	40.7	8	32	58.1	41.9
AMP	≥64	≥64	20.8	79.2	≥64	≥64	24.1	75.9	≥64	≥64	24.3	75.7	≥64	≥64	23.0	77.0
FEP	≤0.5	≥64	60.2	34.0	≤0.5	≥64	61.4	31.8	≤0.5	32	63.3	26.5	≤0.5	≥64	61.5	31.3
CRO	0.12	64	59.9	38.9	0.12	64	61.9	37.6	≤0.06	64	60.6	38.1	0.12	64	61.0	38.1
LVX	8	≥16	43.4	55.4	8	≥16	45.6	54.2	8	≥16	38.1	60.6	8	≥16	43.1	56.1
MEM	≤0.06	0.12	100	0.0	≤0.06	0.12	99.5	0.2	≤0.06	≤0.06	98.7	0.0	≤0.06	0.12	99.5	0.1
MIN	2	16	-	-	1	16	-	-	1	8	-	-	1	8	-	-
TZP	2	32	84.0	11.4	2	64	83.2	12.4	2	16	88.9	7.5	2	32	84.8	11.0
TGC	0.12	0.5	100	0.0	0.12	0.5	97.4	0.0	0.12	0.5	99.6	0.0	0.12	0.5	98.8	0.0
***Enterobacter*** **spp.**																
	*N* = 268				*N* = 389				*N* = 190				*N* = 847			
AMK	2	4	96.6	1.5	2	4	97.4	1.0	2	4	96.3	2.1	2	4	96.9	1.4
FEP	≤0.5	16	66.0	19.4	≤0.5	16	71.2	16.7	≤0.5	16	72.6	15.3	≤0.5	16	69.9	17.2
CRO	1	64	51.5	47.0	0.5	64	58.4	38.8	0.5	64	54.7	40.5	0.5	64	55.4	41.8
LVX	0.06	8	78.0	19.8	0.06	≥16	77.1	21.1	0.06	≥16	83.7	15.8	0.06	≥16	78.9	19.5
MEM	≤0.06	0.5	96.6	0.7	≤0.06	0.25	96.1	1.5	≤0.06	0.25	97.4	0.5	≤0.06	0.25	96.6	1.1
MIN	2	8	-	-	2	8	-	-	2	8	-	-	2	8	-	-
TZP	4	128	57.8	36.9	2	128	65.8	24.9	2	128	67.9	26.8	4	128	63.8	29.2
TGC	0.5	2	89.2	4.5	0.5	2	90.0	4.9	0.5	2	86.8	6.8	0.5	2	89.0	5.2
***Serratia marcescens***																
	*N* = 107				*N* = 131				*N* = 74				*N* = 312			
AMK ^a^	2	8	92.5	4.7	2	4	96.2	3.1	2	8	94.6	0.0	2	8	94.6	2.9
FEP	≤0.5	4	84.1	9.3	≤0.5	1	90.8	3.8	≤0.5	2	89.2	4.1	≤0.5	2	88.1	5.8
CRO	0.25	32	76.6	19.6	0.25	4	86.3	11.5	0.25	8	79.7	17.6	0.25	16	81.4	15.7
LVX	0.12	2	88.8	9.3	0.12	1	94.7	4.6	0.12	0.5	93.2	2.7	0.12	1	92.3	5.8
MEM	0.12	1	93.5	4.7	0.12	0.25	98.5	0.8	≤0.06	0.12	98.6	0.0	0.12	0.25	96.8	1.9
MIN	4	8	-	-	4	8	-	-	2	4	-	-	4	4	-	-
TZP	2	16	86.0	9.3	1	4	92.4	5.3	1	32	82.4	10.8	1	16	87.8	8.0
TGC	1	2	78.5	4.7	1	2	74.8	1.5	1	2	85.1	0.0	1	2	78.5	2.2
***Acinetobacter baumannii***																
	*N* = 182				*N* = 183				*N* = 107				*N* = 472			
AMK	≥128	≥128	14.3	84.1	≥128	≥128	11.5	86.9	≥128	≥128	15.0	79.4	≥128	≥128	13.3	84.1
FEP	32	≥64	-	-	≥64	≥64	-	-	32	≥64	-	-	≥64	≥64	-	-
CAZ	32	32	-	-	32	32	-	-	32	32	-	-	32	32	-	-
CRO	64	64	-	-	64	64	-	-	64	64	-	-	64	64	-	-
LVX	≥16	≥16	7.1	92.9	≥16	≥16	4.4	95.6	≥16	≥16	4.7	94.4	≥16	≥16	5.5	94.3
MEM	≥32	≥32	12.6	84.6	≥32	≥32	5.5	94.5	≥32	≥32	7.5	87.9	≥32	≥32	8.7	89.2
MIN	8	16	-	-	8	16	-	-	4	16	-	-	4	16	-	-
TZP	≥256	≥256	-	-	≥256	≥256	-	-	≥256	≥256	-	-	≥256	≥256	-	-
TGC	0.5	2	-	-	1	2	-	-	0.5	2	-	-	0.5	2	-	-
***Pseudomonas aeruginosa***																
	*N* = 268				*N* = 298				*N* = 173				*N* = 739			
AMK	4	32	79.9	15.3	4	16	87.6	7.4	4	32	81.5	12.7	4	32	83.4	11.5
FEP	8	32	65.3	34.7	4	16	69.8	30.2	8	32	72.8	27.2	8	32	68.9	31.1
CAZ	4	32	70.1	29.9	2	32	76.5	23.5	2	32	78.0	22.0	4	32	74.6	25.4
LVX	2	≥16	47.0	41.4	1	≥16	58.1	31.9	1	≥16	51.4	41.0	1	≥16	52.5	37.5
MEM	1	≥32	59.7	26.5	1	16	72.5	17.1	1	≥32	59.5	27.2	1	≥32	64.8	22.9
TZP	8	128	67.2	32.8	4	128	72.8	27.2	8	64	75.1	24.9	8	128	71.3	28.7
TGC	8	16	-	-	8	16	-	-	8	16	-	-	8	16	-	-
***Haemophilus influenzae***																
	*N* = 98				*N* = 101				*N* = 72				*N* = 271			
AMK	4	16	-	-	4	8	-	-	4	8	-	-	4	8	-	-
AMC	0.5	2	98.0	2.0	0.5	1	99.0	1.0	0.5	2	95.8	4.2	0.5	1	97.8	2.2
AMP	≤0.5	1	91.8	8.2	≤0.5	1	91.1	8.9	≤0.5	4	84.7	15.3	≤0.5	2	89.7	10.3
FEP	≤0.5	≤0.5	-	-	≤0.5	≤0.5	-	-	≤0.5	1	-	-	≤0.5	≤0.5	-	-
CRO	≤0.06	≤0.06	98.0	2.0	≤0.06	≤0.06	98.0	2.0	≤0.06	0.12	91.7	8.3	≤0.06	≤0.06	96.3	3.7
LVX	0.015	0.03	100	0.0	0.015	0.015	100	0.0	0.015	0.25	98.6	1.4	0.015	0.03	99.6	0.4
MEM	≤0.06	0.25	100	0.0	≤0.06	0.25	100	0.0	0.12	0.25	100	0.0	≤0.06	0.25	100	0.0
MIN	≤0.5	1	98.0	0.0	≤0.5	1	96.0	0.0	≤0.5	1	98.6	1.4	≤0.5	1	97.4	0.4
TZP	≤0.06	≤0.06	-	-	≤0.06	≤0.06	-	-	≤0.06	0.12	-	-	≤0.06	≤0.06	-	-
TGC	0.12	0.25	-	-	0.12	0.25	-	-	0.12	0.25	-	-	0.12	0.25	-	-

MIC_50_, minimum inhibitory concentration required to inhibit growth of 50% of isolates (mg/L); MIC_90_, minimum inhibitory concentration required to inhibit growth of 90% of isolates (mg/L); R, resistance; S, susceptibility; AMK, amikacin; AMC, amoxicillin/clavulanate; AMP, ampicillin; FEP, cefepime; CAZ, ceftazidime; CRO, ceftriaxone; LVX, levofloxacin; MEM, meropenem; MIN, minocycline; TZP, piperacillin/tazobactam; TGC, tigecycline; *N* = total number of isolates. ^a^ *S. marcescens* produces a chromosomal AAC(6′)-Ic enzyme that affects the activity of clinically available aminoglycosides, except streptomycin, gentamicin and arbekacin. “-” no EUCAST resistance or susceptibility breakpoints available.

**Table 2:** Rates of Gram-positive and Gram-negative phenotypes collected from Italy between 2012 and 2014.

	2012		2013		2014		2012–2014	
	*n*/*N*	%	*n*/*N*	%	*n*/*N*	%	*n*/*N*	%
**Gram-negative**								
ESBL-*K. pneumoniae*	57/297	19.2	68/304	22.4	41/154	26.6	166/755	22.0
MDR *K. pneumoniae*	136/297	45.8	89/304	29.3	57/154	37.0	282/755	37.4
ESBL-*K. oxytoca*	1/54	1.9	1/73	1.4	0/26	0.0	2/153	1.3
ESBL-*E. coli*	108/332	32.5	121/428	28.3	78/226	34.5	307/986	31.1
MDR *A. baumannii*	140/182	76.9	157/183	85.8	81/107	75.7	378/472	80.1
MDR *P. aeruginosa*	69/268	25.7	41/298	13.8	33/173	19.1	143/739	19.4
βLPos *H. influenzae*	6/98	6.1	8/101	7.9	9/72	12.5	23/271	8.5
BLNAR *H. influenzae*	2/98	2.0	1/101	1.0	2/72	2.8	5/271	1.8
**Gram-positive**								
Methicillin-resistant *S. aureus*	114/331	34.4	163/398	41.0	85/214	39.7	362/943	38.4
Van-R *E. faecalis*	2/142	1.4	6/170	3.5	2/76	2.6	10/388	2.6
Van-R *E. faecium*	13/57	22.8	6/68	8.8	16/53	30.2	35/178	19.7
Pen-R *S. pneumoniae*	1/92	1.1	3/81	3.7	2/72	2.8	6/245	2.4

ESBL, extended-spectrum β-lactamase; MDR, multidrug-resistant; βLPos, β-lactamase positive; BLNAR, β-lactamase negative ampicillin resistant; Van-R, vancomycin-resistant; Pen-R, penicillin-resistant. *N* = total number of isolates; *n* = number of resistant isolates.

**Table 3:** Antimicrobial activity among resistant phenotypes of Gram-negative organisms collected from Italy between 2012 to 2014 ^a^.

	2012				2013				2014				2012–2014			
	MIC_50_	MIC_90_	%S	%R	MIC_50_	MIC_90_	%S	%R	MIC_50_	MIC_90_	%S	%R	MIC_50_	MIC_90_	%S	%R
**ESBL-*Klebsiella pneumoniae***																
	*N* = 57				*N* = 68				*N* = 41				*N* = 166			
AMK	4	32	61.4	17.5	4	16	83.8	4.4	4	16	82.9	7.3	4	16	75.9	9.6
AMC	32	≥64	5.3	94.7	16	≥64	10.3	89.7	16	≥64	17.1	82.9	32	≥64	10.2	89.8
FEP	≥64	≥64	8.8	87.7	≥64	≥64	2.9	95.6	32	≥64	2.4	97.6	≥64	≥64	4.8	93.4
CRO	64	64	0.0	98.2	64	64	0.0	100	64	64	2.4	97.6	64	64	0.6	98.8
LVX	≥16	≥16	15.8	82.5	≥16	≥16	13.2	85.3	8	≥16	12.2	87.8	≥16	≥16	13.9	84.9
MEM	0.25	≥32	64.9	29.8	0.12	≥32	75.0	22.1	≤0.06	≥32	80.5	19.5	0.12	≥32	72.9	24.1
MIN	4	8	-	-	4	8	-	-	4	8	-	-	4	8	-	-
TZP	≥256	≥256	21.1	70.2	16	≥256	35.3	44.1	16	≥256	41.5	43.9	32	≥256	31.9	53.0
TGC	1	2	82.5	7.0	1	2	73.5	8.8	1	2	70.7	9.8	1	2	75.9	8.4
**MDR *Klebsiella pneumoniae***																
	*N* = 136				*N* = 89				*N* = 57				*N* = 282			
AMK	16	32	11.0	45.6	16	≥128	24.7	38.2	16	32	29.8	21.1	16	32	19.1	38.3
AMC	≥64	≥64	3.7	96.3	≥64	≥64	3.4	96.6	≥64	≥64	5.3	94.7	≥64	≥64	3.9	96.1
FEP	≥64	≥64	3.7	94.9	≥64	≥64	3.4	95.5	≥64	≥64	5.3	94.7	≥64	≥64	3.9	95.0
CRO	64	64	4.4	95.6	64	64	4.5	95.5	64	64	5.3	94.7	64	64	4.6	95.4
LVX	≥16	≥16	0.0	100	≥16	≥16	0.0	100	≥16	≥16	0.0	100	≥16	≥16	0.0	100
MEM	≥32	≥32	9.6	89.7	≥32	≥32	11.2	86.5	≥32	≥32	14.0	86.0	≥32	≥32	11.0	87.9
MIN	4	8	-	-	4	16	-	-	4	8	-	-	4	8	-	-
TZP	≥256	≥256	4.4	95.6	≥256	≥256	4.5	93.3	≥256	≥256	5.3	91.2	≥256	≥256	4.6	94.0
TGC	1	2	69.9	7.4	1	4	57.3	15.7	1	4	52.6	15.8	1	4	62.4	11.7
**ESBL-*Escherichia coli***																
	*N* = 108				*N* = 121				*N* = 78				*N* = 307			
AMK	4	8	94.4	0.9	4	8	93.4	2.5	4	16	87.2	3.8	4	8	92.2	2.3
AMC	16	32	28.7	71.3	16	32	44.6	55.4	16	32	34.6	65.4	16	32	36.5	63.5
AMP	≥64	≥64	0.0	100	≥64	≥64	0.0	100	≥64	≥64	1.3	98.7	≥64	≥64	0.3	99.7
FEP	32	≥64	2.8	88.0	32	≥64	1.7	83.5	16	≥64	7.7	66.7	32	≥64	3.6	80.8
CRO	64	64	0.0	98.1	64	64	2.5	96.7	64	64	1.3	97.4	64	64	1.3	97.4
LVX	8	≥16	7.4	92.6	8	≥16	5.8	94.2	8	≥16	5.1	93.6	8	≥16	6.2	93.5
MEM	≤0.06	0.12	100	0.0	≤0.06	0.12	99.2	0.8	≤0.06	0.12	97.4	0.0	≤0.06	0.12	99.0	0.3
MIN	2	16	-	-	1	8	-	-	1	8	-	-	2	8	-	-
TZP	4	32	75.9	16.7	4	64	79.3	12.4	2	64	78.2	12.8	4	64	77.9	14.0
TGC	0.12	0.5	100	0.0	0.12	0.5	98.3	0.0	0.12	0.5	98.7	0.0	0.12	0.5	99.0	0.0
**MDR *Acinetobacter baumannii***																
	*N* = 140				*N* = 157				*N* = 81				*N* = 378			
AMK	≥128	≥128	0.0	100	≥128	≥128	0.0	100	≥128	≥128	0.0	100	≥128	≥128	0.0	100
FEP	≥64	≥64	-	-	≥64	≥64	-	-	≥64	≥64	-	-	≥64	≥64	-	-
CAZ	32	32	-	-	32	32	-	-	32	32	-	-	32	32	-	-
CRO	64	64	-	-	64	64	-	-	64	64	-	-	64	64	-	-
LVX	≥16	≥16	0.0	100	≥16	≥16	0.0	100	≥16	≥16	0.0	100	≥16	≥16	0.0	100
MEM	≥32	≥32	0.0	100	≥32	≥32	0.0	100	≥32	≥32	0.0	100	≥32	≥32	0.0	100
MIN	8	16	-	-	8	16	-	-	8	16	-	-	8	16	-	-
TZP	≥256	≥256	-	-	≥256	≥256	-	-	≥256	≥256	-	-	≥256	≥256	-	-
TGC	1	2	-	-	1	2	-	-	1	2	-	-	1	2	-	-
**MDR *Pseudomonas aeruginosa***																
	*N* = 69				*N* = 41				*N* = 33				*N* = 143			
AMK	32	≥128	36.2	50.7	8	64	51.2	43.9	32	64	30.3	60.6	32	64	39.2	51.0
FEP	32	≥64	13.0	87.0	16	≥64	4.9	95.1	16	≥64	18.2	81.8	16	≥64	11.9	88.1
CAZ	32	32	27.5	72.5	32	32	24.4	75.6	16	32	42.4	57.6	16	32	30.1	69.9
LVX	≥16	≥16	2.9	95.7	≥16	≥16	2.4	95.1	≥16	≥16	0.0	100	≥16	≥16	2.1	96.5
MEM	≥32	≥32	10.1	87.0	16	≥32	7.3	87.8	16	≥32	9.1	87.9	16	≥32	9.1	87.4
TZP	64	≥256	15.9	84.1	64	≥256	14.6	85.4	32	128	27.3	72.7	64	≥256	18.2	81.8
TGC	16	16	-	-	16	16	-	-	16	16	-	-	16	16	-	-

MIC_50_, minimum inhibitory concentration required to inhibit growth of 50% of isolates (mg/L); MIC_90_, minimum inhibitory concentration required to inhibit growth of 90% of isolates (mg/L); R, resistance; S, susceptibility; AMK, amikacin; AMC, amoxicillin/clavulanate; AMP, ampicillin; FEP, cefepime; CAZ, ceftazidime; CRO, ceftriaxone; LVX, levofloxacin; MEM, meropenem; MIN, minocycline; TZP, piperacillin/tazobactam; TGC, tigecycline; ESBL, extended-spectrum β-lactamase; MDR, multidrug-resistant; *N* = total number of isolates. ^a^ Data not shown for ESBL-*K. oxytoca*, β-lactamase positive *H. influenzae* and β-lactamase negative ampicillin-resistant *H. influenzae* as the number of isolates submitted was <10 in each year of collection. “-” no EUCAST resistance or susceptibility breakpoints available.

**Table 4:** Antimicrobial activity among Gram-positive organisms collected in Italy between 2012 and 2014.

	2012				2013				2014				2012–2014			
	MIC_50_	MIC_90_	%S	%R	MIC_50_	MIC_90_	%S	%R	MIC_50_	MIC_90_	%S	%R	MIC_50_	MIC_90_	%S	%R
**Methicillin susceptible *Staphylococcus aureus***																
	*N* = 217				*N* = 235				*N* = 129				*N* = 581			
AMC	1	2	-	-	0.5	2	-	-	1	2	-	-	1	2	-	-
AMP	2	8	-	-	2	16	-	-	1	8	-	-	2	16	-	-
CRO	2	4	-	-	2	4	-	-	2	4	-	-	2	4	-	-
LVX	0.25	4	87.1	12.0	0.25	0.5	94.5	5.1	0.12	0.5	93.0	6.2	0.12	1	91.4	7.9
LZD	2	2	100	0.0	2	2	100	0.0	2	2	100	0.0	2	2	100	0.0
MEM	0.25	0.5	-	-	≤0.12	0.25	-	-	≤0.12	0.25	-	-	≤0.12	0.25	-	-
MIN	≤0.25	0.5	96.8	1.4	≤0.25	≤0.25	98.3	1.7	≤0.25	≤0.25	95.3	3.9	≤0.25	≤0.25	97.1	2.1
PEN	2	≥16	18.9	81.1	2	≥16	24.7	75.3	2	≥16	24.0	76.0	2	≥16	22.4	77.6
TZP	0.5	1	-	-	0.5	1	-	-	0.5	1	-	-	0.5	1	-	-
TGC	0.12	0.12	100	0.0	0.12	0.12	100	0.0	0.12	0.12	100	0.0	0.12	0.12	100	0.0
VAN	0.5	1	100	0.0	0.5	1	100	0.0	0.5	1	100	0.0	0.5	1	100	0.0
***Enterococcus faecalis***																
	*N* = 142				*N* = 170				*N* = 76				*N* = 388			
AMP ^a^	1	2	99.3	0.0	1	1	100	0.0	0.5	2	100	0.0	1	2	99.7	0.0
LVX	1	≥64	-	-	1	≥64	-	-	1	≥64	-	-	1	≥64	-	-
LZD	1	2	100	0.0	2	2	100	0.0	1	2	100	0.0	2	2	100	0.0
MEM	4	8	-	-	4	8	-	-	4	8	-	-	4	8	-	-
MIN	8	8	-	-	8	8	-	-	8	8	-	-	8	8	-	-
PEN	2	8	-	-	2	8	-	-	2	4	-	-	2	8	-	-
TZP	2	8	-	-	2	8	-	-	2	8	-	-	2	8	-	-
TGC	0.06	0.12	100	0.0	0.12	0.12	100	0.0	0.06	0.12	100	0.0	0.06	0.12	100	0.0
VAN	1	2	98.6	1.4	1	2	96.5	3.5	1	2	97.4	2.6	1	2	97.4	2.6
***Enterococcus faecium***																
	*N* = 57				*N* = 68				*N* = 53				*N* = 178			
AMP ^a^	≥32	≥32	5.3	93.0	≥32	≥32	17.6	80.9	≥32	≥32	15.1	84.9	≥32	≥32	12.9	86.0
LVX	≥64	≥64	-	-	≥64	≥64	-	-	≥64	≥64	-	-	≥64	≥64	-	-
LZD	2	2	100	0.0	2	2	100	0.0	2	2	100	0.0	2	2	100	0.0
MEM	≥32	≥32	-	-	≥32	≥32	-	-	≥32	≥32	-	-	≥32	≥32	-	-
MIN	4	8	-	-	1	8	-	-	4	8	-	-	4	8	-	-
PEN	≥16	≥16	-	-	≥16	≥16	-	-	≥16	≥16	-	-	≥16	≥16	-	-
TZP	≥32	≥32	-	-	≥32	≥32	-	-	≥32	≥32	-	-	≥32	≥32	-	-
TGC	0.06	0.06	100	0.0	0.06	0.12	98.5	1.5	0.06	0.25	100	0.0	0.06	0.12	99.4	0.6
VAN	1	≥64	77.2	22.8	1	2	91.2	8.8	1	≥64	69.8	30.2	1	≥64	80.3	19.7
***Streptococcus pneumoniae***																
	*N* = 92				*N* = 81				*N* = 72				*N* = 245			
AMC	≤0.03	0.12	-	-	≤0.03	1	-	-	≤0.03	0.5	-	-	≤0.03	0.25	-	-
AMP	≤0.06	0.25	95.7	1.1	≤0.06	1	88.9	3.7	≤0.06	1	87.5	5.6	≤0.06	0.5	91.0	3.3
AZI	0.12	64	55.6	44.4	0.12	≥128	64.1	33.3	0.12	64	72.9	27.1	0.12	64	63.4	35.7
CRO	≤0.03	0.25	96.7	0.0	≤0.03	0.5	93.8	0.0	≤0.03	1	87.5	0.0	≤0.03	0.5	93.1	0.0
CLI	0.03	64	55.6	44.4	0.03	≥128	61.5	38.5	0.03	≥128	72.9	27.1	0.03	≥128	62.6	37.4
CLN	0.03	≥128	64.4	35.6	0.06	≥128	70.5	29.5	0.06	≥128	74.3	25.7	0.06	≥128	69.3	30.7
ERY	0.03	64	56.7	43.3	0.03	≥128	64.1	35.9	0.06	≥128	72.9	27.1	0.03	64	63.9	36.1
LVX	1	1	100	0.0	1	1	95.1	4.9	0.5	1	100	0.0	1	1	98.4	1.6
LZD	≤0.5	1	100	0.0	≤0.5	1	100	0.0	≤0.5	1	100	0.0	≤0.5	1	100	0.0
MEM	≤0.12	0.25	100	0.0	≤0.12	0.25	100	0.0	≤0.12	0.5	100	0.0	≤0.12	0.25	100	0.0
MIN	1	8	43.5	31.5	1	8	32.1	32.1	0.5	4	56.9	26.4	1	8	43.7	30.2
PEN	≤0.06	0.25	64.1	1.1	≤0.06	0.5	66.7	3.7	≤0.06	1	76.4	2.8	≤0.06	0.5	68.6	2.4
TZP	≤0.25	≤0.25	-	-	≤0.25	1	-	-	≤0.25	2	-	-	≤0.25	1	-	-
TGC	0.015	0.03	-	-	0.015	0.03	-	-	0.015	0.03	-	-	0.015	0.03	-	-
VAN	0.25	0.5	100	0.0	0.25	0.5	100	0.0	0.25	0.5	100	0.0	0.25	0.5	100	0.0
***Streptococcus agalactiae***																
	*N* = 111				*N* = 125				*N* = 80				*N* = 316			
AMC	0.06	0.12	-	-	0.06	0.12	-	-	0.06	0.12	-	-	0.06	0.12	-	-
AMP	≤0.06	0.12	-	-	0.12	0.12	-	-	≤0.06	0.12	-	-	0.12	0.12	-	-
CRO	0.06	0.12	-	-	0.06	0.12	-	-	0.06	0.12	-	-	0.06	0.12	-	-
LVX	0.5	1	96.4	3.6	0.5	1	96.0	2.4	0.5	1	100	0.0	0.5	1	97.2	2.2
LZD	1	1	100	0.0	1	1	100	0.0	1	1	100	0.0	1	1	100	0.0
MEM	≤0.12	≤0.12	-	-	≤0.12	≤0.12	-	-	≤0.12	≤0.12	-	-	≤0.12	≤0.12	-	-
MIN	8	8	17.1	82.0	8	≥16	12.8	86.4	8	8	12.5	83.8	8	8	14.2	84.2
PEN	≤0.06	0.12	100	0.0	0.12	0.12	100	0.0	≤0.06	≤0.06	100	0.0	≤0.06	0.12	100	0.0
TZP	≤0.25	≤0.25	-	-	≤0.25	≤0.25	-	-	≤0.25	≤0.25	-	-	≤0.25	≤0.25	-	-
TGC	0.03	0.06	100	0.0	0.03	0.03	100	0.0	0.03	0.06	100	0.0	0.03	0.06	100	0.0
VAN	0.25	0.5	100	0.0	0.5	0.5	100	0.0	0.5	0.5	100	0.0	0.5	0.5	100	0.0

MIC_50_, minimum inhibitory concentration required to inhibit growth of 50% of isolates (mg/L); MIC_90_, minimum inhibitory concentration required to inhibit growth of 90% of isolates (mg/L); R, resistance; S, susceptibility; AMK, amikacin; AMC, amoxicillin-clavulanate; AMP, ampicillin; AZI, azithromycin; FEP, cefepime; CRO, ceftriaxone; CLI, clarithromycin; CLN, clindamycin; ERY, erythromycin; LVX, levofloxacin; LZD, linezolid; MEM, meropenem; MIN, minocycline; PEN, penicillin; TZP, piperacillin-tazobactam; TGC, tigecycline; VAN, vancomycin; *N* = total number of isolates. ^a^ Susceptibility to amoxicillin with and without β-lactamase inhibitor can be inferred from ampicillin [8]. “-” no EUCAST resistance or susceptibility breakpoints available.

**Table 5:** Antimicrobial activity among resistant phenotypes of Gram-positive organisms collected from Italy between 2012 to 2014 ^a^.

	2012				2013				2014				2012–2014			
	MIC_50_	MIC_90_	%S	%R	MIC_50_	MIC_90_	%S	%R	MIC_50_	MIC_90_	%S	%R	MIC_50_	MIC_90_	%S	%R
**Methicillin-resistant *Staphylococcus aureus***																
	*N* = 114				*N* = 163				*N* = 85				*N* = 362			
AMC	8	≥16	-	-	8	≥16	-	-	4	≥16	-	-	8	≥16	-	-
AMP	16	≥32	-	-	16	≥32	-	-	16	≥32	-	-	16	≥32	-	-
CRO	32	≥128	-	-	64	≥128	-	-	16	≥128	-	-	32	≥128	-	-
LVX	16	≥64	14.0	84.2	16	32	16.6	82.2	16	≥64	12.9	84.7	16	≥64	14.9	83.4
LZD	2	2	100	0.0	2	2	100	0.0	2	2	100	0.0	2	2	100	0.0
MEM	4	≥32	-	-	4	≥32	-	-	2	≥32	-	-	4	≥32	-	-
MIN	≤0.25	≤0.25	95.6	3.5	≤0.25	0.5	96.9	1.8	≤0.25	≤0.25	98.8	1.2	≤0.25	≤0.25	97.0	2.2
PEN	≥16	≥16	0.0	100	≥16	≥16	0.0	100	8	≥16	0.0	100	≥16	≥16	0.0	100
TZP	16	≥32	-	-	16	≥32	-	-	8	≥32	-	-	16	≥32	-	-
TGC	0.12	0.25	100	0.0	0.12	0.25	100	0.0	0.12	0.12	100	0.0	0.12	0.25	100	0.0
VAN	0.5	1	100	0.0	0.5	1	100	0.0	0.5	1	100	0.0	0.5	1	100	0.0
**Vancomycin-resistant *Enterococcus faecium***																
	*N* = 13				*N* = 6				*N* = 16				*N* = 35			
AMP ^b^	≥32	≥32	0.0	100	≥32	≥32	[0]	[6]	≥32	≥32	0.0	100	≥32	≥32	0.0	100
LVX	≥64	≥64	-	-	≥64	≥64	-	-	≥64	≥64	-	-	≥64	≥64	-	-
LZD	2	2	100	0.0	2	2	[6]	[0]	2	2	100	0.0	2	2	100	0.0
MEM	≥32	≥32	-	-	≥32	≥32	-	-	≥32	≥32	-	-	≥32	≥32	-	-
MIN	4	8	-	-	1	8	-	-	2	8	-	-	4	8	-	-
PEN	≥16	≥16	-	-	≥16	≥16	-	-	≥16	≥16	-	-	≥16	≥16	-	-
TZP	≥32	≥32	-	-	≥32	≥32	-	-	≥32	≥32	-	-	≥32	≥32	-	-
TGC	0.06	0.25	100	0.0	0.06	0.12	[6]	[0]	0.03	0.12	100	0.0	0.06	0.12	100	0.0
VAN	≥64	≥64	0.0	100	32	≥64	[0]	[6]	≥64	≥64	0.0	100	≥64	≥64	0.0	100

MIC_50_, minimum inhibitory concentration required to inhibit growth of 50% of isolates (mg/L); MIC_90_, minimum inhibitory concentration required to inhibit growth of 90% of isolates (mg/L); R, resistance; S, susceptibility; AMC, amoxicillin/clavulanate; AMP, ampicillin; CRO; ceftriaxone; LVX, levofloxacin; LZD, linezolid; MEM, meropenem; MIN, minocycline; PEN, penicillin; TZP, piperacillin/tazobactam; TGC, tigecycline; VAN, vancomycin. ^a^ Data not shown for vancomycin-resistant *Enterococcus faecalis* and penicillin-resistant *Streptococcus pneumoniae* as the number of resistant isolates submitted was <10 in each year of collection. ^b^ Susceptibility to amoxicillin with and without beta-lactamase inhibitor can be inferred from ampicillin [8]. Percentage susceptible or resistant not calculated when <10 isolates. In these cases total number of isolates susceptible to resistant are given in square brackets. “-” no EUCAST resistance or susceptibility breakpoints available.
